# When a bank becomes a bank, and a bank is the bank but not the bank: Multistability of homonyms’ meaning

**DOI:** 10.1177/20416695231194210

**Published:** 2023-08-27

**Authors:** Malin Styrnal, Claus-Christian Carbon, Alexander Pastukhov

**Affiliations:** General Psychology and Methodology, 14310 Otto-Friedrich-Universitat Bamberg, Bamberg, Germany

**Keywords:** rivalry/bistability, audition, cognition, listening, perception

## Abstract

Perceptual multistability is well-known and mostly visually demonstrated: Common examples are Necker's cube or Rubin's face-vase that produce qualitatively different percepts continuously oscillating between the solutions despite physically stable stimuli. We lack knowledge about similar phenomena in other domains, for instance in linguistics, where we are faced with homonyms that create multistability of cognitive semantics, differently assigned meanings of identical words. Our participants listened to repeated presentations of homonyms for which two or even three meanings could be assigned, and they reported the dominant meaning perceived at a certain point in time. Results showed that most participants experienced multistability of meaning for homonyms, with semiperiodic changes in dominant meaning similar to multistabity in perception. These findings suggest that multistability is a general property of the brain's neural architecture that resolves ambiguity irrespective of the level of representation.

Our perception of the outside world is constructed from intrinsically ambiguous and noisy sensory inputs combined with knowledge about statistical regularities of the world (Metzger, 2009). One striking example of this is multistable perception produced by stimuli compatible with several comparably probable interpretations. This balanced ambiguity produces alternations in perception despite a physically constant stimulus. What is remarkable about multistability is that it can be evoked for different modalities and levels of representation. In vision, binocular rivalry displays lead to the suppression of monocular inputs at the level of the primary visual cortex (Tong et al., 2006) and even modulates activity in subcortical structures such as lateral geniculate nucleus (Wunderlich et al., 2005). Yet, similar switching perception can be induced with respect to visual depth (Necker cube, Schroeder stairs), three-dimensional motion (kinetic-depth effect), and figure versus ground perception (Rubin's vase). Beyond the visual empire, we detect this kind of switching for auditory (Deutsch, 1974; Noorden, 1975; Warren & Gregory, 1958), olfactory (Zhou & Chen, 2009), tactile (Holcombe & Seizova-Cajic, 2008), and proprioceptive perception (Seizova-Cajic et al., 2007). All of these alternations take place despite stimuli being physically constant.

Building on the multistability of perception, one can construct ambiguous images that produce changes in both perception and meaning, as in classic old-young woman (Donaldson, 2017) or duck-rabbit (Donaldson, 2016) drawings. But can we take it even further to produce multistability of *meaning* alone, while the physical stimulus and the respective sensory signal remain constant? We looked at the ambiguity of homonyms, words with the same spelling and pronunciation, i.e., words that are both homographs and homophones. For example, in English, “the bank” could refer to a financial institution, but also to a riverbank, and “rock” could refer to a stone and a music genre. We used 20 German homonyms plus five control items with just a single dominant meaning, that is, nonhomonyms (Table 1). The study consisted of 25 trials (one trial for each word) and a test run. In each trial, we presented an audio recording of the respective word that was repeated 30 times over a period of approximately one minute. Participants had to indicate the perceived meaning and could also report “None” if they perceived no meaning, as semantic satiation can occur when hearing a word repeatedly (Jakobovits & Lambert, 1962; Wetherill, 2014). Twenty-two participants completed the experiment, see https://doi.org/10.17605/OSF.IO/JM8GE for open methods, data, and analysis.

**Table 1.** A selection of words used in the study, their assumed meaning(s), and translation to English (in blue). Word frequencies are reported on a scale from 1 (rare) to 6 (frequent) (DWDS, 2023).

Word (German)	(A) Words with a single meaning (German | English)									Word frequency
Brief	geschriebene Nachricht					letter				4
Gedicht	lyrische Dichtung					poem				3
Word	(B) Words with two meanings (German | English)									
Schloss	Palast		castle		Verschluss			lock		4
Sprosse	Querholz einer Leiter		rung		Pflanzentrieb			sprout		2
Kiefer	Kieferknochen		jaw		Baum			pine		3
Word	(C) Words with three meanings (German | English)									
Messen	Maß nehmen	to measure		Gottesdienste			masses	Ausstellung	fairs	4
Decken	Federbetten	blanket		Zimmerdecken			ceilings	Tisch decken	set the table	3

We found that the repetition of homonyms induced switching between different dominant meanings, similar to alternations in perception during visual multistability, see Figure 1. Words differed greatly in the relative dominance of meaning, with words like *Absatz* or *Decken* showing balanced bi- and tristability, whereas words like *Ball* or *Gut* effectively had a single dominant meaning. The individual dominance phases were long even for balanced bistable stimuli with a single phase lasting for dozens of seconds.

**Figure 1.**
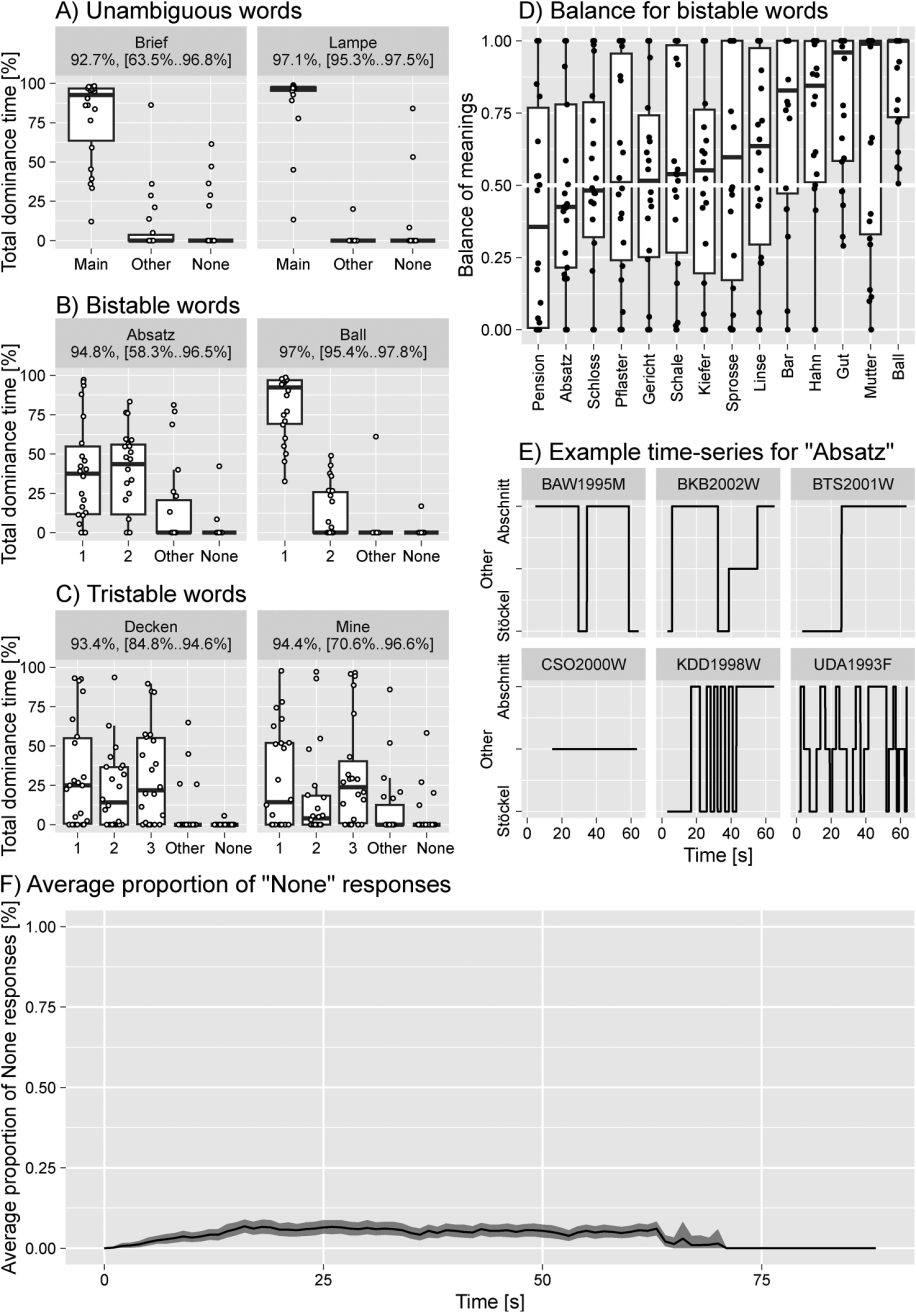
Results. The proportion of time individual meanings were reported for words with (A) one, (B) two, and (C) three dominant meanings. Dots depict individual participants. The labels above the plot show the median proportion and the interquartile range for the total dominance of primary meaning(s) together. (D) The relative dominance of meaning #1 for words with two primary meanings, dots depict individual participants. (E) Example time-series of dominance reports for the word *Absatz*. (F) The average proportion of “None” responses over all participants and words (black line) and the 97% bootstrapped confidence interval (shade).

Our results extend the list of multistable domains by demonstrating that the processing of homonyms shows similar effects to well-documented multistable stimuli. This provides further support for the idea that multistability is a general property of the brain's neural architecture that resolves any kind of ambiguity. For visual stimuli, this competition can occur at various levels of visual hierarchy, in our case of cognitive semantics, competition occurs at the cognitive level of words’ meaning. However, conceptually similar mechanisms have been postulated at the level of decision-making both for perceptual decisions (Gold & Shadlen, 2007) and for delay discounting decisions (Scherbaum et al., 2016).

Taken together, this reinforces the idea that multistability is not a perceptual phenomenon per se but reflects computations of local but architecturally similar circuits that resolve ambiguity and support decision-making in a volatile world (Cao et al., 2021). Viewed from this angle, perceptual multistability induced by visual stimuli is merely a most convenient way to study the underlying neural mechanisms, rather than being an end in themselves. And, as the phenomenon of multistability is linked to the architecture of underlying circuits, it should manifest itself whenever we are able to supply balanced ambiguous inputs for such a circuit. In the present work, we employed words with different meanings, but further examples are likely to be discovered.

We are looking forward to receiving ideas or reports soon!
